# Bisphenol A—A Dangerous Pollutant Distorting the Biological Properties of Soil

**DOI:** 10.3390/ijms222312753

**Published:** 2021-11-25

**Authors:** Magdalena Zaborowska, Jadwiga Wyszkowska, Agata Borowik, Jan Kucharski

**Affiliations:** Department of Soil Science and Microbiology, University of Warmia and Mazury in Olsztyn, Plac Łódzki 3, 10-727 Olsztyn, Poland; m.zaborowska@uwm.edu.pl (M.Z.); agata.borowik@uwm.edu.pl (A.B.); jan.kucharski@uwm.edu.pl (J.K.)

**Keywords:** BPA, soil enzymes, soil microorganisms, biodiversity, spring rape, maize

## Abstract

Bisphenol A (BPA), with its wide array of products and applications, is currently one of the most commonly produced chemicals in the world. A narrow pool of data on BPA–microorganism–plant interaction mechanisms has stimulated the following research, the aim of which has been to determine the response of the soil microbiome and crop plants, as well as the activity of soil enzymes exposed to BPA pressure. A range of disturbances was assessed, based on the activity of seven soil enzymes, an abundance of five groups of microorganisms, and the structural diversity of the soil microbiome. The condition of the soil was verified by determining the values of the indices: colony development (CD), ecophysiological diversity (EP), the Shannon–Weaver index, and the Simpson index, tolerance of soil enzymes, microorganisms and plants (TI_BPA_), biochemical soil fertility (BA_21_), the ratio of the mass of aerial parts to the mass of plant roots (PR), and the leaf greenness index: Soil and Plant Analysis Development (SPAD). The data brought into sharp focus the adverse effects of BPA on the abundance and ecophysiological diversity of fungi. A change in the structural composition of bacteria was noted. Bisphenol A had a more beneficial effect on the *Proteobacteria* than on bacteria from the phyla *Actinobacteria* or *Bacteroidetes*. The microbiome of the soil exposed to BPA was numerously represented by bacteria from the genus *Sphingomonas*. In this object pool, the highest fungal OTU richness was achieved by the genus *Penicillium*, a representative of the phylum *Ascomycota*. A dose of 1000 mg BPA kg^−1^ d.m. of soil depressed the activity of dehydrogenases, urease, acid phosphatase and *β*-glucosidase, while increasing that of alkaline phosphatase and arylsulfatase. Spring oilseed rape and maize responded significantly negatively to the soil contamination with BPA.

## 1. Introduction

The chemical structure of bisphenol A (2,2-bis(4-hydroxyphenyl)propane) with two phenol groups substituted by hydroxyl groups in the para position provides the chemical with good reactivity associated with electrophilic substitution or transformation into ethers, esters and salts [1,2]. Commercial production and use of bisphenol A (BPA) began in the USA in 1957 and a year later it was launched in Europe [3]. The global volume of BPA produced exceeded 4.6 × 10^9^ kg in 2012 and continues to increase at an annual rate of 4.6%, thus predicted to reach an output of 7.4 × 10^9^ kg in 2023 [4]. It is foreseen that the annual BPA production or import within the European Economic Area will reach 10.6 × 10^9^ kg in 2022 [5]. This trend corresponds with the worldwide growing demand for food and beverage packaging, medical equipment, electronic devices, building materials, and paper coatings, in which BPA is a significant plasticizer [6]. BPA as a synthetic xenoestrogen is an intermediate or final component of important consumer products, such as detergents, phenolic resins, epoxy resins, polycarbonates, polyesters and polyacrylates [7]. According to the report issued by the European Food Safety Authority [8], thermal paper is the second-largest source of exposure to BPA, after foodstuff. Hence, its production on the EU market, which has been maintained on a stable level of 1–10 × 10^9^ kg since 2017, encouraged the establishment of the BPA concentration threshold at 0.02% by weight, which came into force in January 2020 [9]. In turn, Canada and France in 2010, Argentina and Brazil in 2012 and Belgium in 2013, legally banned the use of BPA in baby bottles [10]. These measures are a consequence of numerous reports on the toxic effect of bisphenols on the human body. First and foremost, BPA is a compound that strongly destabilizes the endocrine system classified as an endocrine-disrupting chemical (EDC) [11]. Gingrich et al. [12] report that exposure to this phenolic compound during pregnancy has an adverse effect on the development of the fetus. The placental tissue was found to contain 273.9 ng BPA g^−1^ [13]. Ahbab et al. [14] prove that BPA affects the expression of steroidogenic enzymes and testosterone production in Leydig cells. Studies dealing with autism spectrum disorders reveal another hazardous aspect of bisphenol due to its interference with synaptogenesis and neurogenesis processes [15]. Some 90% of BPA is removed from blood through the kidneys and excreted in urine as PBA-G [16,17]. The broad array of applications of BPA has resulted in its frequent presence in various environmental compartments, such as surface waters, sewage, sediments, groundwater and soil [18]. As the highest average annual BPA production increase occurs in India, where it is linked to a 19% increase in the demand for polycarbonates, the surface waters in this country have been determined to contain high concentrations of BPA, reaching 7.2 μg BPA dm^−3^ [19,20]. The consequences of the economic development strategies implemented in the United States of America, Germany or Italy have been just as serious [21]. Noteworthy is the fact that BPA is detected in tap water, in which it reacts with hypochlorite (ClO) ions, or in water used for hemodialysis [22]. According to Choi and Lee [23], sewage sludge is a considerable reservoir of bisphenols. The content of bisphenols in this environmental matrix oscillates from 10 to 100,000 μg kg^−1^ d.m. of sludge. According to the probabilistic distribution of the risk of contaminating sewage sludge with bisphenol A, the quantity of this pollutant is approximately 14,200 μg kg^−1^ d.m. of sewage in North America, and 95,000 μg kg^−1^ d.m. of sewage in Europe [24]. These statistical data are essential because the main sources of soil contamination with bisphenol A are sewage sludge [25], discharged leachate from landfills, and irrigation with wastewater [26]. Of importance is also the recycling and disposal of electronic waste [27]. The transport of organic pollutants in groundwater is significantly affected by the soil content of organic matter, responsible for sorption, complexing and ion exchange of bisphenols [28]. These processes are induced by the synergy of hydrophobic interactions and formation of hydrogen bonds with soil minerals [29]. Diagboy et al. [30] maintain that iron oxides also play a key role in the sorption of bisphenol A in soil. Nevertheless, degradation of bisphenols positively correlated with soil pH proceeds more rapidly in soils (t1/2 = 30–360) than in sewage sludge (t1/2 = 135–1621 days) [21]. This is the outcome of the activity of microorganisms which induce the expression of various genes located in chromosomes or in plasmids. Franchi et al. [31] demonstrate that during the anaerobic degradation of phenols via the 4-hydroxybenzoate to benzoyl-CoA pathway, the cleavage of the 6-oxycyclohex-1-endo-1-carbonyl-CoA ring is catalyzed by hydrolase encoded on the bamA gene. Phenolic hydroxylase encoded by the pheA2A1 gene and regulated by the PheR gene (family AraC) participates in the first stage of phenol degradation by conducting hydroxylation of the aromatic ring [32]. The PheR gene was identified in *Acinetobacter calcoaceticus* NCIMB 8250 and in *Pseudomonas pseudoalcaligenes* NCIMB 9867 [33,34]. A similar function is performed by the ncgl2587 gene of *Corynebacterium glutamicum*, which belongs to the family of AraC/XylS transcription regulators and has been characterized by Chen et al. [35]. Molds also partake in BPS degradation owing to the activity of peroxidases responsible for the oxidation of lignins and laccases catalyzing the oxidation, decarboxylation and demethylation of polyphenols [36]. Although microorganisms display potential for biodegradation, the growth of many strains is inhibited by bisphenols [37], which is demonstrated in studies based on molecular methods concerning both the genotype and phenotype diversity [38]. Thus far, the activity of soil enzymes submitted to BPA pressure has not been investigated on such a broad scale, which is puzzling in view of the fact that they are recognized as mediators of the soil’s metabolic potential and reliable indicators of its biological quality, nowadays indispensable in the planning of sustainable management practices [39,40,41,42].

Of fundamental importance in any assessment of the scale of BPA toxicological effects is an analysis of complex interactions of this phenolic compound with plants. According to Dodgen et al. [43], the highest potential for the uptake by plants is possessed by chemical compounds whose octanol-water partition coefficient (logK_ow_) oscillates around 1 up to 3.5. The hydrophobicity of BPA has been estimated at logK_ow_ = 3.40, which is associated with moderate adsorption and bioaccumulation of this bisphenol [44]. Nonetheless, BPA invariably shows the highest rate of bioconcentration in the roots, and then in the stems and leaves of plants [45]. It is so because the BPA hydroxyl group reacts with ions or binary compounds, such as sulfates and glucuronic acid in soil, forming estrogens which alter the mobility of BPA in the soil-plant system [46]. Moreover, BPA is rapidly metabolized in plant tissues, either by glycosylation or the synthesis of highly polar polymer products [44].

Importantly, dehydrogenases, secreted by both microorganisms and plant roots, oxidize extracellularly the toxic phenolic metabolites through the reduction of electron acceptors NAD +/NADP + [47]. Similarly, both microorganisms and plants are equipped with cytochrome P450 monooxygenases. These enzymes increase the resistance of plants and soil microorganisms to organic pollutants [48], which gives to better understanding of BPA–microorganisms–plant interaction mechanisms. The absence of a broad pool of data constituting a matrix for the emerging evaluation of the scale of a potential inhibitory effect of BPA on the functioning of the soil microbiome’s structural diversity and on the biochemical activity of soil encouraged us to expand the research objective by including an analysis of the impact of this bisphenol on the growth and development of spring oilseed rape and maize. Thus, the aim of this study has been to determine the effect of BPA on the activity of soil enzymes, the abundance and biodiversity of microorganisms, and the response of the test plants to the applied xenobiotic.

## 2. Results

### 2.1. Enzyme Activity

**Figure 1:**
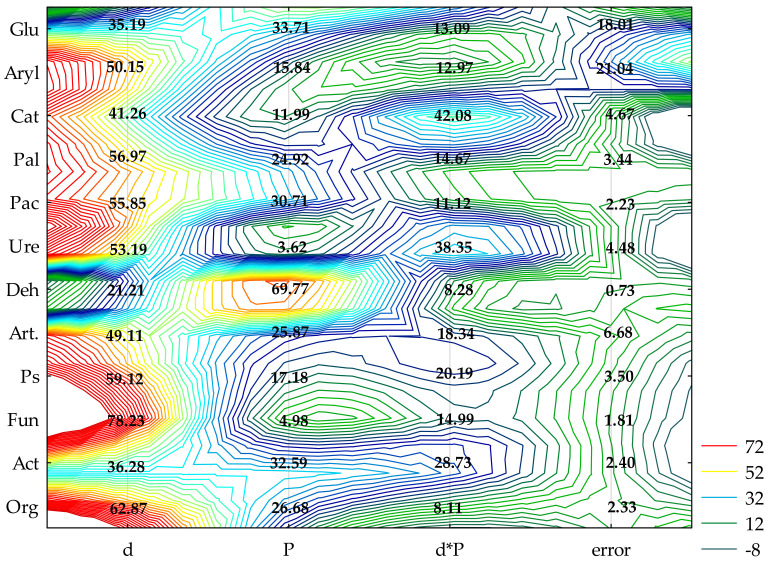
The share of independent variables in the evolution of the enzymes and microorganism activity (η^2^): d—dose of BPA; P—plants; Org—organotrophic bacteria, Act—actinomycetes, Fun—mold fungi, Ps—*Pseudomonas* sp., Art—*Arthrobacter* sp., Deh—dehydrogenases; Cat—catalase; Ure—urease; Pal—alkaline phosphatase; Pac—acid phosphatase; Glu—*β*-glucosidase; Aryl—arylsulfatase (two-way analysis of variance, ANOVA, at *p* < 0.05).

**Figure 2:**
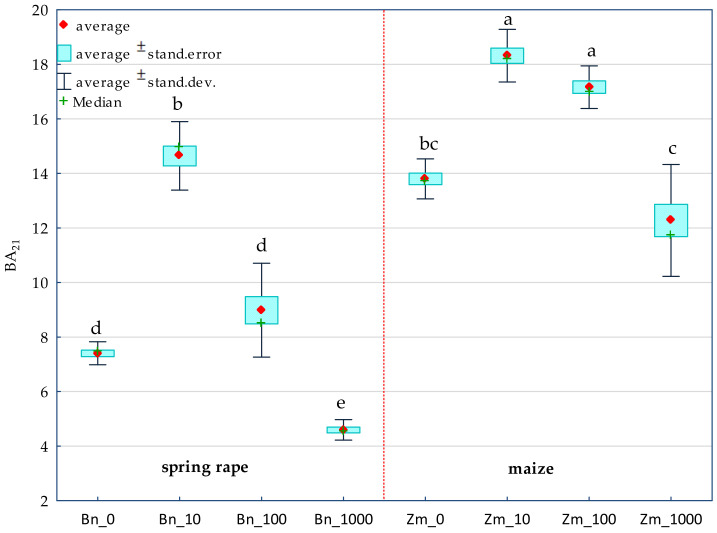
Biochemical fertility index (BA_21_) of soil. BPA dose in mg kg^−1^ d.m. of soil: 0; 10; 100; 1000; Bn—spring rape, Zm—maize; Homogeneous groups denoted with letters (a–e) were calculated separately for each BA_21_ index value in the soil sown with plants.

The achieved values of $\eta^{2}$ show that the increasing levels of soil contamination with BPA had the greatest moderating effect on the activity of alkaline phosphatase (Pal) (57%), while the cropping of soil with spring rape (Bn) and maize (Zm) affected the activity of dehydrogenases (Deh) (68%) to the highest degree (Figure 1). In soil free from BPA, maize raised the activity of Deh and acid phosphatase (Pac) to a much higher degree than spring oilseed rape did. In this pool of objects, these parameters were observed to have increased by 61 and 16%, respectively (Table 1 and Table S1). In the soil submitted to the pressure of 1000 mg BPA kg^−1^ d.m. of soil and cropped with spring oilseed rape, Deh, Pac and urease (Ure) proved to be particularly sensitive to the applied phenolic compound. The compilation of the highest degree of soil contamination with BPA and spring oilseed rape cultivation resulted in an elevated activity of Pal and arylsulfatase (Aryl). The inhibitory effect of BPA in objects seeded with maize was much weaker. However, the application of 1000 mg BPA kg^−1^ d.m. of soil contributed to the inhibition of the activity of Ure (68%) and Deh (11%) relative to control samples (Table S1). The tolerance index TI confirmed the sensitivity of particular enzymes to exposure to BPA (Table 1). Values of TI revealed that, catalase (Cat), Pal and Aryl were the most stable enzymes in soil exposed to BPA contamination, regardless of the species of a plant. Deh and Pac proved to be the least stable. It needs to be underlined that the highest dose of soil pollution with BPA (1000 mg kg^−1^ d.m. of soil) induced a two-fold higher TI value for Deh in soil cropped with maize. The observed tendencies also correspond with the values of the biochemical soil fertility index (BA_21_) obtained in the study (Figure 2). Noteworthy is the positive response of enzymes to the application of 10 mg BPA kg^−1^ d.m. of soil, which contributed to the improvement of soil quality in pots sown with spring oilseed rape (BA_21_ = 14.63) and with maize (BA_21_ = 18.31) compared to the pots not contaminated with BPA.

### 2.2. Counts and Diversity of Microorganisms

The biotic stress caused by BPA soil contamination caused different responses among the individual groups of microorganisms (Figure 1). This is evidenced by the values of $\eta^{2}$, based on which the following series was developed: fungi (Fun) (78%) > organotrophic bacteria (Org) (63%) > *Pseudomonas* sp. (Ps) (59%) > *Arthrobacter* sp. (Art) (49%) > actinomycetes (Act) (36%). The cultivated crop species changed the abundance of microorganisms to a smaller extent. Act (33%) proved to be especially sensitive to the effect produced by the plants. However, when soil not contaminated with BPA was cropped with maize, the abundance of organotrophic bacteria doubled, that of *Pseudomonas* sp. more than trebled, and *Arthrobacter* sp. were almost five-fold more numerous than in the parallel treatments with spring oilseed rape (Table S2). In this pool of objects, the application of 1000 mg BPA kg^−1^ d.m. of soil had a stimulating influence on all groups of microorganisms. However, the cultivation of maize on soil with 10 mg BPA kg^−1^ d.m. of soil decreased the number of organotrophic bacteria, *Pseudomonas* sp. and *Arthrobacter* sp. by 41, 22 and 19%, respectively, relative to the control objects. On the other hand, the compilation of soil contamination with 1000 mg BPA kg^−1^ d.m. of soil and the cropping of soil with spring oilseed rape had an inhibitory effect on the abundance of fungi. This parameter was observed to have declined by 32% in comparison with the uncontaminated samples. The TI of microorganisms towards BPA also highlighted the sensitivity of fungi to the increasing pressure of the phenolic compound irrespective of which plant species was cultivated, as well as strong stimulation of the multiplication of *Pseudomonas* sp. and *Arthrobacter* sp. (Table 2). Values of TI correlated with the dependences obtained in this study demonstrated lesser tolerance of all groups of microorganisms, except fungi, to the exposure to 10 mg BPA kg^−1^ d.m. of soil compared to the higher doses of this xenobiotic polluting the soil.

**Figure 3:**
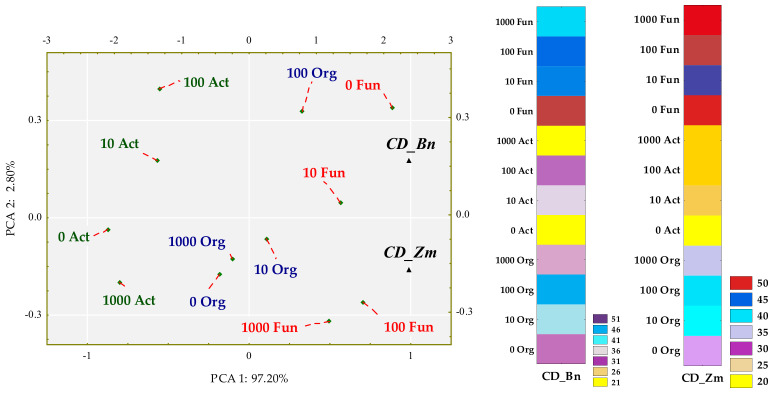
Influence of BPA on the colony development index (CD) of organotrophic bacteria (Org), actinomycetes (Act) and fungi in soil sown with spring rape (Bn) and maize (Zm); ▲—the end of the vector of the primary variable; • cases; doses of BPA: 0; 10; 100; 1000 mg kg^−1^ d.m. of soil.

**Figure 4:**
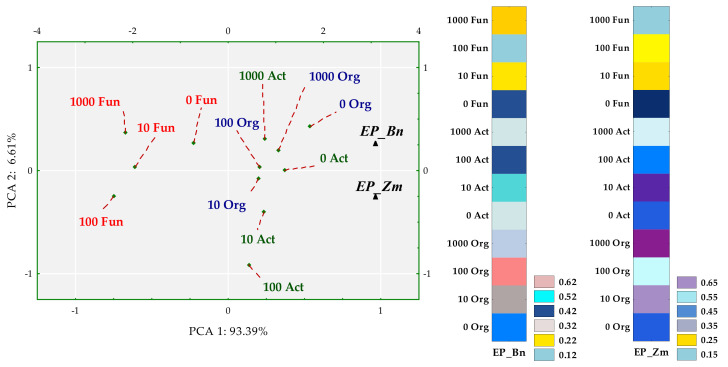
Influence of BPA on the ecophysiological diversity (EP) of organotrophic bacteria (Org), actinomycetes (Act) and fungi in soil sown with spring rape (Bn) and maize (Zm); ▲—the end of the vector of the primary variable; • cases; doses of BPA: 0; 10; 100; 1000 mg kg^−1^ d.m. of soil.

Colony development (CD) and ecophysiological diversity (EP) indices were associated with degrees of soil contamination with BPA and with the plant species. The emerged tendencies were interpreted with multidimensional PCA (Figure 3 and Figure 4).

The first principal component explaining 97.20% of the total variance of data clustered the tips of standardized vectors corresponding to the CD values of spring oilseed rape (Zn) and maize (Zm) (Figure 3). Values of both the coordinates of cases and distances between them revealed a few significant dependences. First of all, the application of BPA to soil did not have a significant inhibitory effect on the multiplication rate of any microbial group. Sowing the soil with maize was not conducive to the multiplication of actinomycetes. In turn, spring rape inhibited the multiplication of fungi in soil contaminated with 1000 mg BPA kg^−1^ d.m. of soil. However, the highest CD values obtained for fungi displayed the potential of this group of microorganisms, in contrast to actinomycetes, to multiply rapidly regardless of being exposed to the phenolic compound. Nevertheless, fungi proved to be sensitive to BPA, which depressed the ecophysiological diversity of this group of microorganisms (Figure 4). The dislocation of cases indicates that the inhibitory strength of the applied xenobiotic towards EP was approximately the same in soil under maize and under spring oilseed rape. It is worth noticing that soil from the pots cropped with maize was found to yield higher EP values for actinomycetes and organotrophic bacteria.

**Figure 5:**
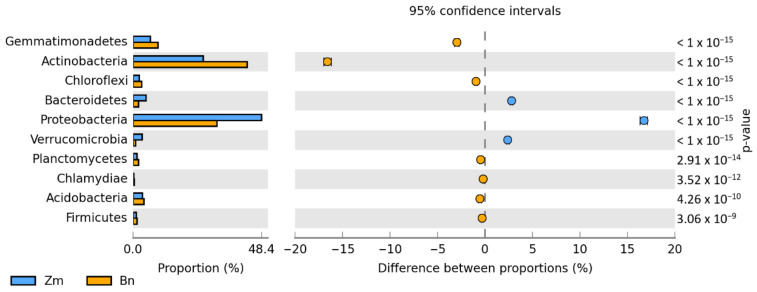

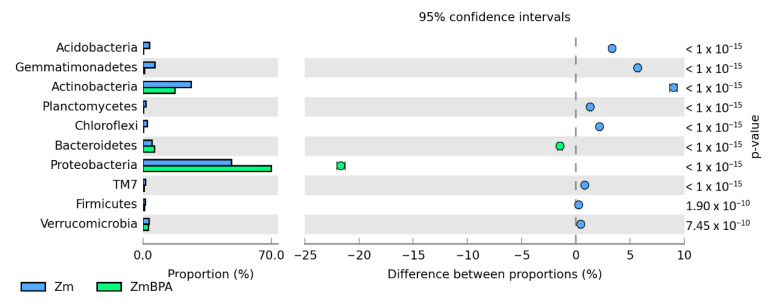

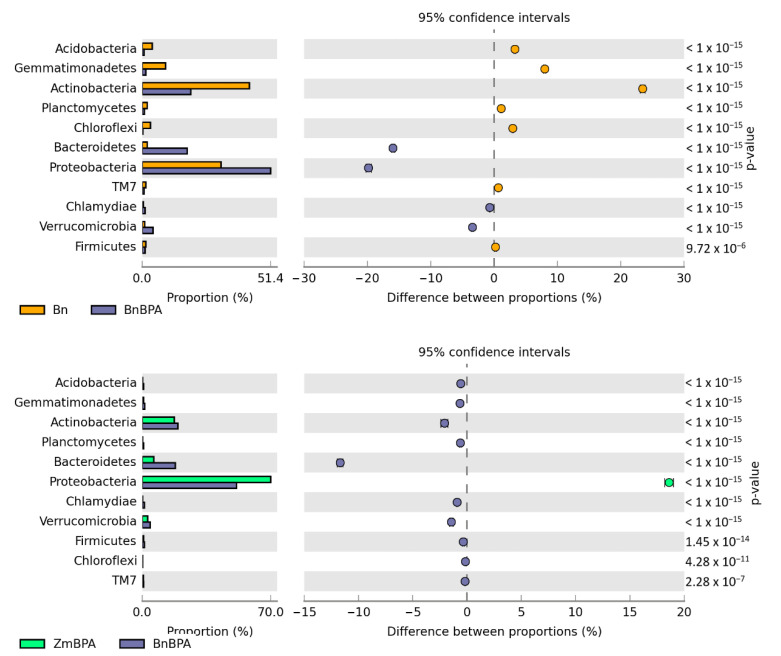
Relative abundance of the dominant types of bacteria in the soil with a difference between proportions ≥ 1%. Zm—maize; Bn—spring rape; BPA—soil contaminated with BPA.

Irrespective of the plant species grown and soil contamination with BPA, bacteria of the phyla *Proteobacteria*, *Actinobacteria* and *Bacteroidetes* proved to be most abundant (Figure 5). Maize stimulated more of an increase in the OTU richness of *Proteobacteria*, while spring oilseed rape, that of *Actinobacteria*. In soil samples taken from maize pots and contaminated with BPA, as much as 70% of OTUs consisted of bacteria classified as *Proteobacteria*. BPA increased the OTU richness of *Proteobacteria* by 21.7% while decreasing the OTU richness of *Actinobacteria* by 9.0%. Similarly, BPA in soil cropped with spring oilseed rape depressed the OTU richness of bacteria from the phylum *Actinobacteria*. The decrease reached as much as 23.5%. BPA in soil cropped with maize increased the OTU richness of bacteria from the phylum *Proteobacteria* in the same order of magnitude (by 19.8%), in addition to which it increased the number of OTUs of bacteria from the phylum *Bacteroidetes* by 15.9%. It should also be emphasized that metagenomic analysis led to the determination of the presence of the *Protista* kingdom represented by the genus *Plasmodiophora* belonging to the *Plasmodiophoromycota* phylum. In soil samples sown with spring oilseed rape (1783 OTU) and maize (966 OTU), the application of BPA to the soil reduced the abundance of *Plasmodiophora* OTU by 76 and 93%, respectively.

**Figure 6:**
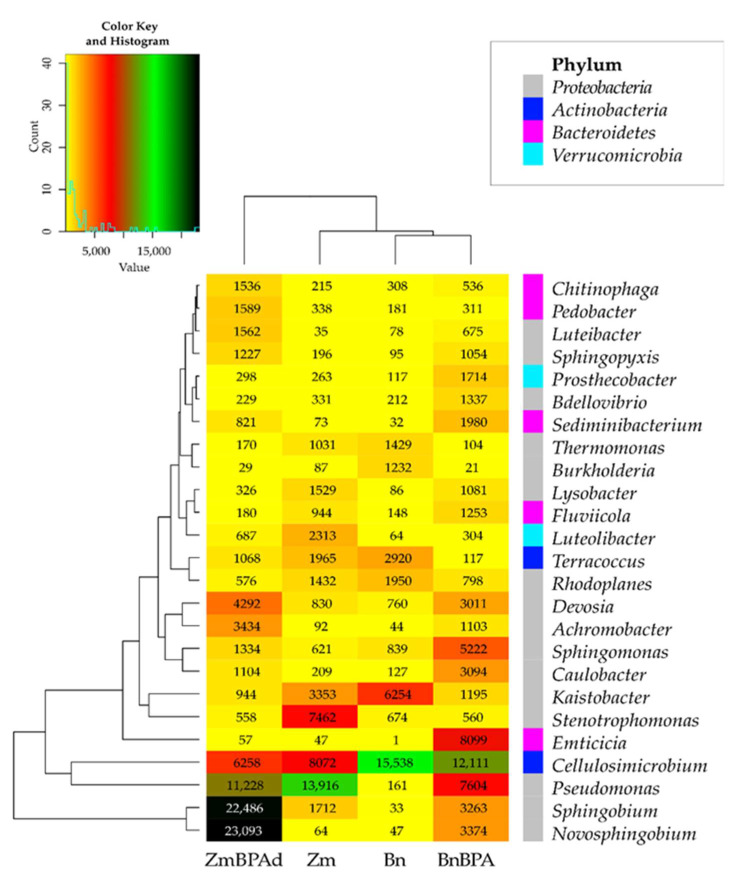
Heat map of the dominant types of bacteria in the soil with a difference between proportions ≥ 1%. Zm—maize; Bn—spring rape; BPA—soil contaminated with BPA.

Changes in the structure of bacteria observed at the level of phyla were reflected in the diversity of bacteria noticed at the level of genera (Figure 6). Samples of soil cropped with spring oilseed rape were dominated by *Cellulosimicrobium* (47%) and *Terracoccus* (9%), assigned to the phylum *Actinobacteria*, and by *Kaistobacter* (19%), from the phylum *Proteobacteria*. In the parallel objects sown with spring oilseed rape, much higher OTU richness was recorded of *Pseudomonas* (13,916) and *Stenotrophomonas* (7462), members of the phylum *Proteobacteria*, representing 29 and 16%, respectively, of all sequences of bacteria at this taxonomic level. Compilation of the BPA soil pollution and the cropping of soil with both plant species led to varied results. Consequences of escalating the stimulatory effect of BPA and spring oilseed rape, which translated into OTU values determined for bacteria, were confirmed for sixteen genera. The highest increase in this parameter following the soil pollution with BPA was determined for *Emticicia*, *Pseudomonas*, *Sphingomonas*. The phenolic compound contributed to a decline in the OTUs of seven genera of bacteria. *Cellulosimicrobium*, *Kaistobacter* and *Terracoccus* proved to be most sensitive.

The reverse tendencies were observed in the pool of pots where the soil was cultivated with maize. BPA had a less beneficial influence on the soil microbiome. While it is true that the application of BPA led to increased OTUs for twelve genera of bacteria, most notably *Novosphingobium* (360–fold higher) and *Sphingobium* (13–fold higher), the BPA pressure depressed the OTU richness for as many as fourteen genera of bacteria, including *Stenotrophomonas* (by 92.5%), *Cellulosimicrobium* (by 22.5%) and *Pseudomonas* (by 19.3%).

**Figure 7:**
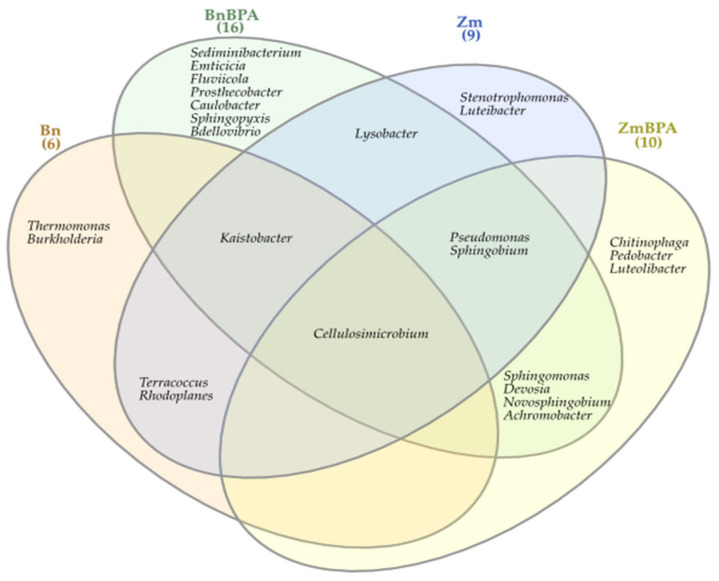
Venn diagram showing unique and common types of bacteria, based on all OTU data; Zm—maize; Bn—spring rape; BPA—soil contaminated with BPA.

**Figure 8:**
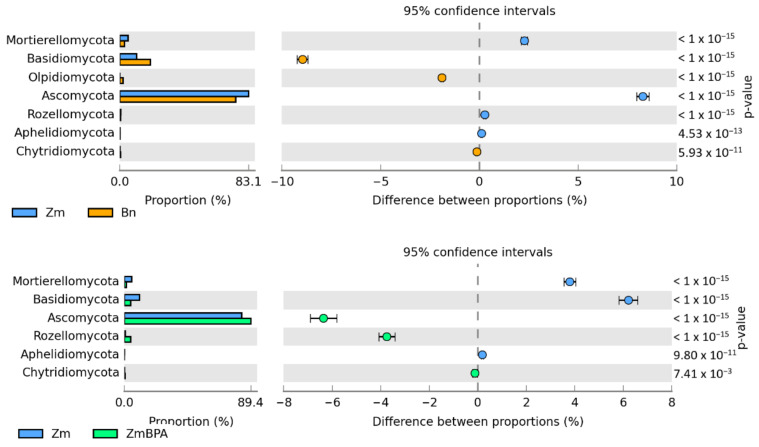

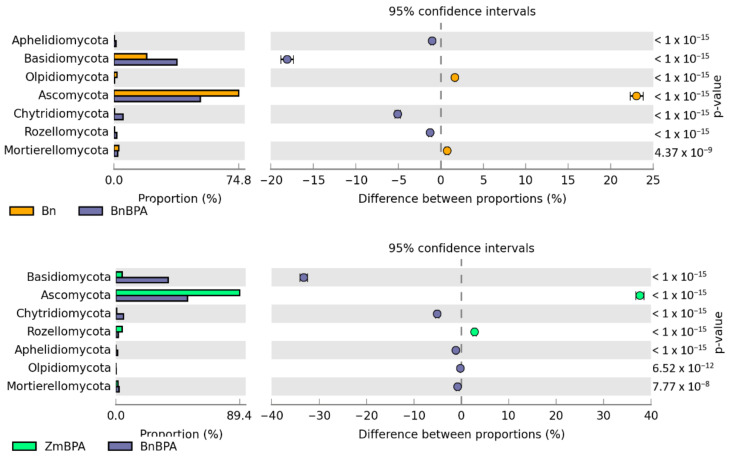
Relative abundance of the dominant types of fungi in the soil with a difference between proportions ≥ 1%. Zm—maize; Bn—spring rape; BPA—soil contaminated with BPA.

The Venn diagram distinguished both shared and unique genera of bacteria representing particular research objects (Figure 7). Taking into account 25 genera of bacteria with an OTU higher than 1% of assigned sequences, it was only the genus *Cellulosimicrobium* which was common for all soil samples, regardless of the grown plant species or pollution. *Terracocus* and *Rhodoplanes* were characteristic for objects not contaminated with BPA, while *Sphingomonas*, *Devosia*, *Novosphingobium* and *Achromobacter* occurred in the pots submitted to BPA pressure. After soil was sown with spring oilseed rape and contaminated with BPA, 16 genera of bacteria were identified in it, of which seven unique taxa were exposed: *Sedimibacterium*, *Emticicia*, *Fluviicola,* all members of the phylum *Bacteroidetes*; *Caulobacter*, *Sphingopyxis*, *Bdellovibrio*, which all belong to the phylum *Proteobacteria*; and *Prosthecobacter*, classified as a member of *Verrucomirobia*. BPA and the two crops interfered significantly with the diversity of mold fungi in soil (Figure 8). According to the OTU values determined at the level of phylum, they were represented by *Ascomycota*. In the soil not polluted with BPA and cropped with maize, *Ascomycota* constituted 83.1% of all mold fungi, which was by 8.3% more than in the pots with spring oilseed rape. The second most abundant moulds were the fungi *Basidiomycota*, which were by 9.0% more abundant in soil under spring oilseed rape than in soil under maize. The phylum *Ascomycota* represented as much as 89.4% of the mold fungi in soil polluted with BPA and cropped with maize, which was by 6.3% more than in unpolluted soil.

**Figure 9:**
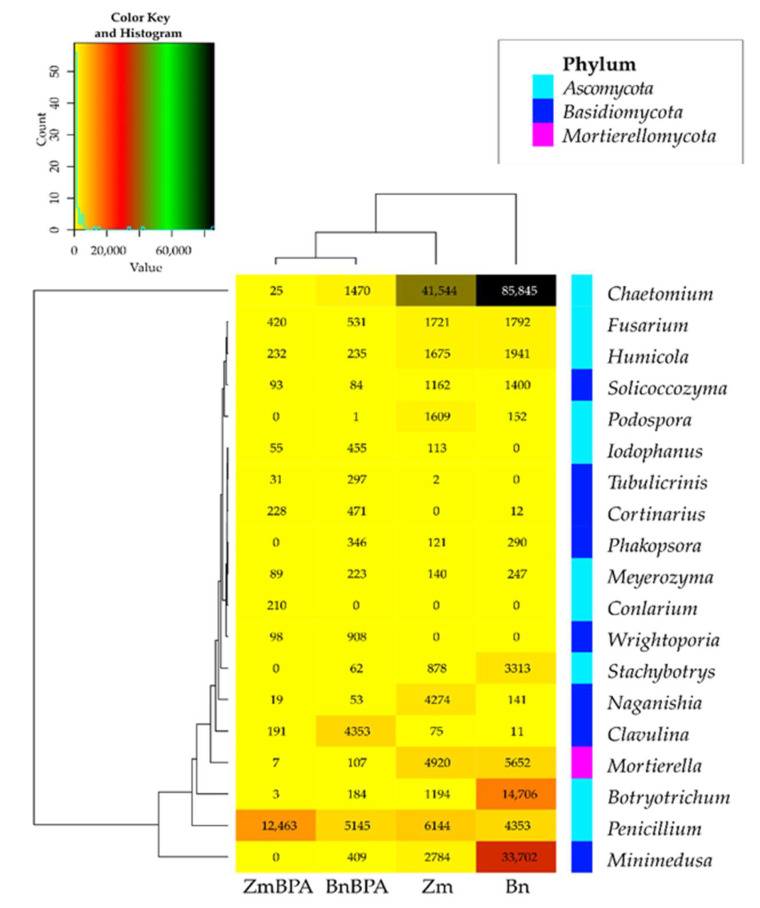
Heat map of the dominant types of fungi in the soil with a difference between proportions ≥ 1%. Zm—maize; Bn—spring rape; BPA—soil contaminated with BPA.

**Figure 10:**
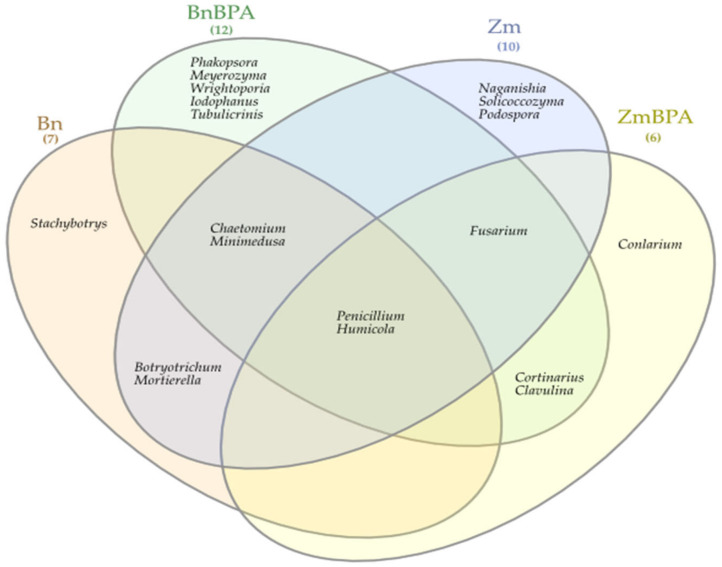
Venn diagram showing unique and common types of fungi, based on all OTU data; Zm—maize; Bn—spring rape; BPA—soil contaminated with BPA.

BPA had an opposite effect on the OTU richness of the fungi *Basidimycota*, as it reduced their abundance by 6.2%. In turn, BPA in soil sown with spring oilseed rape decreased the OTU value of *Ascomycota* by 23.1% while increasing that of fungi of the phylum *Basidiomycota* by 18.1%. Both the species of crops and the soil contamination with BPA differentiated the OTU richness of identified fungal genera, same as in the case of phyla (Figure 9). However, irrespective of a crop species or BPA soil contamination, two representatives of *Ascomycota* were distinguished: *Chaetomium* assigned to the family *Chaetomiaceae*, order *Sordariales* and class *Sordiaromycetes*, and *Penicillium* which belongs to the family *Aspergillaceae*, order *Eurotiales* and class *Eurotiomycetes*. The highest OTU richness of *Chaetomium* was determined in soils free from BPA. BPA decreased the OTUs of *Chaetomium* from 85,845 to 1470 in soil cropped with spring oilseed rape, and from 41,544 to 25 in soil under maize. In turn, *Penicillium* was most numerous in samples of the soil exposed to BPA pressure. Out of 19 identified genera of mold fungi, irrespective of the species of a crop plant, bisphenol A contributed to a decrease in the OTUs of seven mold genera, of which four belonged to the phylum *Ascomycota*: *Chaetomium*, *Botryotrichum*, *Stachybotrys*, *Podospora*, two were classified as members of the phylum *Basidiomycota*: *Minimedusa* and *Soliccocozyma*, and one was a representative of the phylum *Mortierellomycota*: *Mortierella*. It is worth emphasising that BPA had the strongest inhibitory effect on the OTUs of *Chaetomium*. The xenobiotic also induced a rise in the abundance of four genera of mold fungi: *Penicillium* and *Iodophanus*, representing the phylum *Ascomycota*, and *Wrightoporia* and *Tubulicrinis*, which belong to *Basidiomycota*. A significant step in our analyses consisted of tracing the responses of mold fungi to the cropping of soil with the plants and to the BPA pressure by plotting a Venn diagram (Figure 10). It was crucial to distinguish unique genera for particular research objects. Other than maize having a more beneficial effect than spring oilseed rape on the diversity of mold fungi, another finding was that three of the nine representatives of moulds in this pool of objects, were identified as unique ones: *Naganishia*, *Soliccocozyma* and *Podospora*. There was only one unique mold genus, namely *Stachybotrys*, among the seven genera detected in the soil under spring oilseed rape. However, the application of BPA had a much stronger moderating influence on the diversity of moulds. Interestingly, the soil contaminated with bisphenol A and cropped with spring oilseed rape was determined to contain twice as many genera of these fungi (12 genera) as the BPA polluted soil under maize (six genera), and only five unique genera were identified: *Phakopsora*, *Meyerozyma*, *Iodophanus*, *Wrightoporia* and *Tubulicrinis*. A less beneficial effect was noted in soil under maize. There was only one unique genus determined in this treatment, such as *Conlarium*, representing the phylum *Ascomycota*.

### 2.3. The Reaction of Spring Rape and Maize to BPA

**Figure 11:**
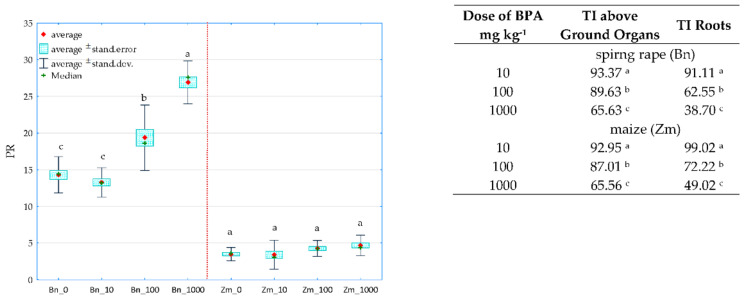
The ratio of the mass of the aerial parts to the mass of plant roots (PR) and the BPA tolerance index of spring rape (Bn) and maize (Zm) (TI); 0, 10, 100, 1000—doses of BPA (mg kg^−1^ d.m. of soil); Homogeneous groups specified in columns denoted with letters (a–c) were calculated separately for each plant, depending on the increasing doses of BPA.

**Figure 12:**
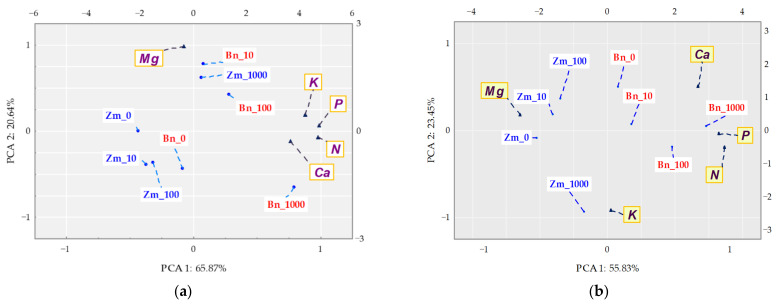
Content of (**a**) and uptake (**b**) of macronutrients by above ground organs of plants (mg kg^−1^ of soil); Zm—maize, Bn—spring rape; ▲—the end of the vector of the primary variable; • cases; doses of BPA: 0; 10; 100; 1000 mg kg^−1^ d.m. of soil.

A holistic dimension of the research was achieved when the response of spring oilseed rape and maize to the increasing levels of soil contamination with bisphenol was traced. BPA added to soil considerably distorted the yield of the crops, which is confirmed by values of the plants’ tolerance index (TI) to this xenobiotic (Figure 11). One of the determined parameters was the ratio of the mass of aerial parts to the mass of roots of plants (PR). A negative correlation was demonstrated between the growing pressure of the phenolic compound and the mass of spring oilseed rape roots, not corresponding to the response of maize. A significant aspect of the verification of plants’ tolerance to the soil contamination with the phenolic compound consisted of analysing the interaction between BPA applied to soil and the content and uptake of macronutrients by spring oilseed rape and by maize (Figure 12). Based on the PCA results, it can be concluded that BPA has increased the content of N, P, K in the plants. However, the phenolic compound had a negative effect on the content of Mg in both spring rape (Figure 12a) and maize (Figure 12b). The distribution of all cases on a PCA map implies a positive correlation between the growing soil pollution with BPA and the content of N, P, K and Ca in the aerial organs of spring rape, but no such relationship was found for Mg. It is additionally confirmed by positive values of the tips of vectors of all basic variables relative to the first main component (PCA1), explaining 65.87% of total variance of the data. In turn, the uptake of macronutrients by plants highlighted the positive effect of the highest BPA dose (1000 mg kg^−1^ d.m. of soil) on the uptake of N, P and K, as well as its inhibitory effect on the uptake of Mg and Ca by maize (Figure 12b). One of the research tasks was to determine the relative chlorophyll content, expressed by the leaf greenness index SPAD (Table S4). No significant influence of BPA on this parameter was observed.

## 3. Discussion

### 3.1. Soil Enzymes

There are many enzymes active in the soil environment playing key roles in biochemical processes [49]. Their efficiency depends on physical and chemical soil properties, such as texture or pH, which affect the sorption, proteolysis and inactivation of enzymes [50]. According to Shuler and Kargi [51], pH can moderate the activity of soil enzymes in three ways: by affecting the affinity of the substrate for a given enzyme, by changing the three-dimensional shape of an enzyme, or by influencing its active site. Thus, although the activity of dehydrogenases suggests the presence of live and physiologically active microorganisms, thereby enabling the control of population changes among soil microorganisms [52], the mentioned dependences were not so obvious. A need, therefore, arose to perform a multifaceted analysis of the research results, which implicated that the response of dehydrogenases to BPA applied to soil was different from the response of microorganisms to this compound. The tendencies demonstrated in this study attest to the results reported by Perotti [53], who maintained that hydroquinone, a toxic intermediate metabolite of phenols, inhibited the activity of dehydrogenases. According to Carvalho et al. [54], this is a response to oxidative stress induced by the pressure of the phenolic compound, whose mechanism relies on the accumulation of nicotinamide adenine dinucleotide (NAD)-dependent lactate dehydrogenase by microorganisms. A dose of 1000 mg BPA kg^−1^ d.m. of soil had an inhibitory effect on the activity of acid phosphatase, urease and *β*-glucosidase. The current research results are comparable to the results obtained by Zaborowska et al. [55,56] and Siczek et al. [57]. The research showed a negative effect of BPA on the biochemical activity of soil. The sensitivity of soil enzymes to the increasing pressure of the phenolic compound was ranked as follows: Deh > Ure > Glu > Pac > Ca t> Aryl > Pal [55]. However, urease and arylsulfatase turned out to be the most sensitive, while dehydrogenases were the least vulnerable to the pressure of 100 mg of bisphenol S (BPS) and bisphenol F (BPF), BPA analogues and the least of [56]. In turn, the negative impact of 500 mg BPS kg^−1^ d.m. of soil on dehydrogenases, acid phosphatase and urease proved to be more severe [58]. Dehydrogenases were the most sensitive to soil contamination with BPF after the application of 5 mg kg^−1^ d.m. of soil [59]. Taking into account the sensitivity of soil enzymes to the pressure of *o*-cresol in doses of 10 and 50 mg kg^−1^ d.m. of soil, they were ranked as follows: CAT > PAC = ARYL > URE > DEH > PAL > GLU [60]. In the research of Zhu et al. [61], exposure to the cresol frothing agent inhibited urease activity as well. However, it needs to be added that the effect of BPA on the biochemical activity of soil is significantly influenced by the elevated sorption of this chemical compound, strictly connected with the cation exchange capacity, content of iron oxides, as well as the content of clays and exchangeable fractions of inorganic fractions in soil [62].

### 3.2. Number and Diversity of Bacteria and Fungi

A complex evaluation of the soil microbiome creates an opportunity to predict the interactions of microorganisms under BPA pressure and to gain better understanding of the functions of soil [63]. However, it should be mentioned that phenolic compounds in the soil structure show a much greater affinity for the humic fraction than for fulvic acids, which is strictly connected with the function of building structural nuclei of humic substances that these phenolic compounds perform [64]. Nonetheless, this does not negate the fact that phenolics are xenobiotics that disrupt the state of soil homeostasis. Probably, the greater sensitivity of bacteria to the lower level of BPA contamination (10 mg kg^−1^ d.m. of soil) observed in this study is due to the toxicity of bisphenols manifested by their interference with the metabolism of purines, pyrimidines and phospholipid fatty acids (PLFAs) [65]. BPA, easily adsorbed on hydrophobic components of the cell surface, binds to the cell membrane by intercalation, thereby changing the membrane permeability, which also affects the expression of amino acids and proteins [66]. In the research by Zaborowska et al. [60], 50 mg of *o*-cresol kg^−1^ d.m. of soil stimulated the multiplication of both organotrophic bacteria and actinomycetes. However, this phenolic compound had a negative impact on the biodiversity of these groups of microorganisms. *O*-cresol also induced an increase in the biodiversity of mold fungi.

Having determined in this study the positive effect of BPA on the abundance of all groups of microorganisms except mold fungi, one might feel inclined to trace their biodegradability potential. *Pseudomonas*, whose abundance increased with the increasing pressure of BPA, can serve as an example. Many reports suggest that the mentioned relationship is associated with the potential of *Pseudomonas*, which are capable of inducing many metabolic pathways by being equipped with a wide pool of genes responsible for the degradation of bisphenols [67]. The following are counted as the most significant ones: gene-encoding halohydrin dehalogenase (HHDH) (*Pseudomonas umsongensis*) [68], gene of phenol 1,2 dioxygenase (*Pseudomonas* sp. PH11), gene of catechol 2,3 dioxygenase (*Pseudomonas* sp. PH7), xyIEJI104 gene (*Pseudomonas aeruginosa* JI104) and nahH gene (*Pseudomonas stutzeri* CLN100) [69]. The PCR analysis has shown that the genes encoding the dioxygenases: catechol 2,3-dioxygenase, catechol 1,2-dioxygenase and protocatechuate 3,4-dioxygenase oxidize catechol or protocatechuate via the α-ketoacid and β-ketoadipate pathways. The three amplified dioxygenase genes indicated that both pathways serve as general mechanisms for the catabolism of catechols or protocatechuate derived from phenolic compounds [70]. Bisphenol A (BPA) is metabolized by a Gram-negative aerobic bacterium via a novel pathway involving the oxidative skeletal rearrangement of BPA. Oxidation of the aliphatic methyl group of BPA leads to coproduction of methyl-hydroxylated 2,2-bis(4-hydroxyphenyl)-1-propanol and a skeletally rearranged triol 1,2-bis(4-hydroxyphenyl)-2-propanol. Eventually, both 4-hydroxybenzoic acid and 4-hydroxyacetophenone are mineralized [71]. The microorganism utilizes (R)-2,3-dichloro-1-propanol, whereas the (S)-enantiomer is not degraded [72]. This is caused by the high enantioselectivity of the halohydrin dehalogenase, the first enzyme in the degradation pathway [73].

Fungi are also distinguished by possessing a wealth of enzymes catalysing the decomposition of BPA. The following are among the most important in this respect: manganese peroxidase (MnP) and laccase, which metabolise bisphenol to phenol, hexestrol, 4-isopropeniphenol and others [74]. Fungi are also able to convert phenolic compounds to seven aromatic intermediate compounds: gentisic acid, gallic acid, protocatechuic acid, catechol, pyrogallol, hydroquinone and hydroxyquinol, and eventually to pyruvate or acetyl-CoA [75]. It is therefore puzzling that 1000 mg BPA kg^−1^ d.m. of soil had an inhibitory effect on the abundance and ecophysiological diversity (EP) of fungi. Similar tendencies were observed by Li et al. [76] and Zaborowska et al. [56]. This might be due to a stronger, negative impact produced on microorganisms by intermediate metabolites of the cleavage of bisphenols, such as acyl halides or hydroquinone [77].

The analysis of the soil microbiome diversity based on nucleotide sequences made it possible to identify representatives of the microbiome of BPA contaminated soil. In our study, microorganisms representing the phyla *Proteobacteria* and *Actinobacteria* were dominant in soil cropped with spring oilseed rape or with maize. However, the bacterial composition changed under the BPA pressure to the advantage of *Proteobacteria*, a finding which agrees with reports published by many researchers [56,57,78]. It is not without a reason that the most numerous representatives of *Proteobacteria* in soils contaminated with BPA were the bacteria of the genus *Sphingomonas*. These are the most frequently isolated microorganisms that degrade the phenolic compound in question [79]. High efficiency of the biodegradation of bisphenols has been attributed to many of its strains, e.g., *Sphingomonas* sp. AO1, *Sphingomonas* sp. MV1 [80], *Sphingomonas* sp. SO11, SO1a, SO4a [81], *Sphingomonas* sp. BP-7 [82] and *Sphingomonas yanoikuyae* BP-11R [83]. This process is induced by the substitution mechanism of ipso-hydroxylation with the participation of monooxygenase supported by FAD. In the subsequent stage, the C-C bond between the BPA phenolic group and the isopropyl group breaks, leading to the formation of hydroquinone degraded to organic acids [37]. The bacteria which can also cometabolize or use bisphenol A as a source of carbon proved to be *Devosia*, *Novosphingobium* and *Achromobacter*. The results of our observations coincide with the pools of microorganisms indicated by Zhang et al. [79] and Zaborowska et al. [55].

Exposure to BPA moderated the communities of both bacteria and fungi. The Shannon–Wiener and the Simpson indices showed that BPA significantly reduced the diversity of bacteria and fungi in soil under maize (Table S3). They also exhibited weaker sensitivity of bacteria and greater sensitivity of fungi to the applied xenobiotic in soil seeded with oilseed rape. Considering the dominance of *Ascomycota* in soil contaminated with BPA, it might be expected that representatives of moulds on the genus level would be the taxa which belong to the mentioned phylum, especially *Penicillium*. According to Shedbalkar’a et al. [84] *Penicillium ochrochlorom* synthesizes lignin peroxidase, which catalyses the one-electron oxidation of phenolic compounds. In the parallel pots, it was also expected to observe escalation of the OTU richness of *Chaetomium* and *Podospora*. According to the report by Mitbaá et al. [85], laccase-producing *Chaetomium strumarium G5I* was particularly efficient at degrading BPA. The response of *Podospora* seemed less debatable. It is known that this fungus is rich in the genes responsible for the synthesis of three types of oxidases included in the pool of laccases: oxidase ABR1, ascorbate oxidase (AO1) and ferroxidase (FET3), oxidizing phenolic compounds [86]. The diminishing OTU richness of *Podospora* under the pressure of BPA was probably caused by the sensitivity of these enzymes to hydroquinone, an intermediate metabolite of bisphenol [87]. Close to the canonical FET3, a group of ABR1-like proteins have been described to act in the DHN (1,8-dihydroxynaphthalene)-melanin synthesis pathway. It involves the de novo synthesis of the phenolic compound DHN by a polyketide synthase (PKS) pathway and the subsequent polymerization, producing the DHN melanin [88]. In turn, ascorbate oxidases (AO) are found in both plants and fungi, in which they catalyze the reduction of oxygen to water, preferentially using ascorbate as an electron donor and leading to the production of monodehydroascorbate [89]. It has been also shown that lower AO expression confers resistance to unfavorable environmental conditions [90].

### 3.3. The Response of Spring Rape and Maize to BPA Pressure

The observed decreasing tolerance of spring oilseed rape and maize to the increasing BPA pressure was justifiable. Research reports point to a wide array of mechanisms induced by plants in response to environmental factors [45,91,92] However, it is eventually the degree of soil contamination with BPA that has a major influence on disturbances in the growth and development of plants [93]. Kang et al. [94] report that high BPA doses lead to the bioaccumulation of this compound and cause toxic effects. The most undesirable effect of BPA toxicity is the inhibited growth of plant roots [95], which was noted in our studies, particularly in the case of spring oilseed rape. This is most probably due to the fact that endogenous hormones: indole-3-acetic acid (IAA), zeatin (ZT) and abscisic acid (ABA), are responsible for the regulation of plant cells in the plant–BPA interaction system [96]. A side effect of soil contamination with BPA is an elevated amount of ABA. Abscisic acid retards the growth of primary and lateral roots and inhibits the synthesis of IAA and ZT, responsible for the growth of roots and leaves [97]. Frejd et al. [98] also discovered that BPA induces the production of reactive forms of oxygen, including H_2_O_2_, in plant roots, which suggests that bisphenol triggers a reaction to oxidative stress. Unquestionably, the main cause of distorted root growth is the damage caused to the matrix of cytoskeletal microtubules on root tips [95]. The results of research by Zhang et al. [91] indicate that ROS (reactive oxygen species) may be involved in the oxidative metabolism of BPA, preventing damage to the exposed plants by this phenolic compound.

The milder response of maize is probably a consequence of inducing the phenylpropanoid pathway by a pool of 10 genes, including two phenylpropanoid genes: PAL9 and 4CL3, six lignin genes: ALDH1, ALDH5, COMT1, F5H1, HCT, HCT10, and two flavonoid genes: FLS1 and C2 [99]. They are important regulators of the germination of maize kernels and their response to biotic and abiotic stresses [100]. It is worth underlining that gene expression in plant cells undergoes regulation for the plants to adjust to the BPA pressure. Detoxification genes of glutathione transferase and UDP-glucosyltransferase, activated as a defence system, are strongly expressed in the presence of BPA. Nevertheless, the application of 45 mg BPA kg^−1^ d.m. of soil in an experiment by Ali et al. [93] led to an elevated activity of such genes as catalase and ascorbate peroxidase, which—according to the researchers—equated with the structural and functional cell damage. The lack of any effect of BPA on the relative chlorophyll content in oilseed rape and in maize was most probably a consequence of the hydrophobic nature of this bisphenol (logKow = 3.40), which resulted in a tendency for weak migration of this xenobiotic to the aerial organs of plants and its accumulation in roots [45]. However, Kim et al. [92] speculated that exposure to BPA (>500 mg kg^−1^ d.m. of soil) disturbed the synthesis of chlorophyll. This was probably due to the reduction in the size of the stomata. Zhang et al. [101] hypothesize that there are differences in the extent to which BPA interferes with the fluorescence of chlorophyll, depending on plant species.

## 4. Materials and Methods

### 4.1. BPA

**Figure 13:**
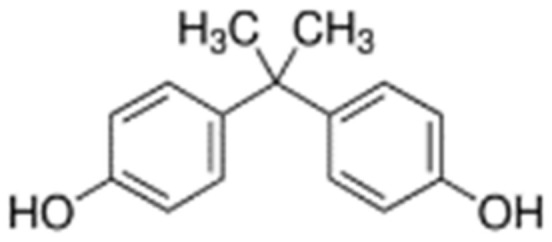
Chemical structure of BPA.

The subject of the research was bisphenol A (BPA) CAS: 80-05-7, 4,4′-isopropylidenediphenol; 2,2-bis(4-hydroxyphenyl)-propane) (Figure 13). According to the Sigma Aldrich (St. Louis, MO, USA) safety data sheet, BPA (C_15_H_16_O_2_) is a white crystalline substance with purity ≥ 98.0% (HPLC). BPA is characterized by the following physicochemical properties: bioconcentration factor (BCF)—71.85, vapor pressure (VP)—2.27 × 10^−7^, water solubility (WS); predicted data (at 25 °C, mg L^−1^)—172.7, boiling point (BP) (°C)—363.5 [102].

### 4.2. Soil Characteristics

Soil was sampled from the northern part of the Olsztyńskie Lake District (NE Poland, 53.72° N, 20.42° E), which lies in the East European Plain in the East Baltic–Belarusian Lowland region and is characterized by the prevalence of soils developed on sands and clays. Before soil sampling, the soil material was used as agricultural soil for the cultivation of varietal plants including *Avena sativa* cv. Bingo (oats), according to the practices suitable for the transitional warm temperate climate zone. The soil selected for the research belongs to Eutric Cambisols. It was sampled from a depth of 0–20 cm. This was loamy sand of the following composition: 74.5% of sand, 22.9% of silt and 2.2% of clay. The physicochemical properties of the soil were as follows: hydrolytic acidity (HAC)—11.4 mM (+) kg^−1^ d.m. of soil; sum of exchangeable base cations (EBC)—49 mM(+) kg^−1^ d.m.; cation exchange capacity (CEC)—60.4 mM (+) kg^−1^ d.m.; base saturation (BS)—81.1 %, pH_KCl_—6.7 and C_org_—9.3 g kg^−1^ d.m. As soil enzymes are considered to be reliable indicators of soil quality, the characterization of soil material was extended by analysing its biochemical and microbiological characteristics, presented in Table 3. All analyses were carried out in accordance with the methods described by Borowik et al. [103].

### 4.3. Experiment Design

Prior to starting the pot experiment, soil was transported to a greenhouse on the campus of the University of Warmia and Mazury in Olsztyn and sieved through a mesh size 1 cm. Batches of 3.5 kg of soil mixed with mineral fertilizers, added in amounts corresponding to the plants’ nutritional requirements, were packed into polyethylene pots. The fertilization was composed of the following forms and doses of nutrients in mg kg^−1^ of soil: nitrogen (N) as CO(NH_2_)_2_—120, phosphorus (P) as KH_2_PO_4_ —40, potassium (K) as KCl and KH_2_PO_4_—120, and magnesium (Mg) as MgSO_4_·7H_2_O —20. The experiment was conducted in four replications, applying increasing doses of BPA to the soil: 0, 10, 100, 1000 mg BPA kg^−1^ d.m. of soil. BPA was added to the soil having been previously dissolved in ethanol in a 3:1 ratio (ethanol:bisphenol). Another experimental variable was the species of the crop. The experiment was set up in 32 pots, of which 16 were cropped with maize (*Zea mays* variety *LG 32.52*), and 16 with spring oilseed rape (*Brassica napus*, variety *Menthal*). The soil material prepared as described above was brought to the soil moisture content of 60% capillary capacity and this level of moisture was maintained throughout the entire plant growing season. After harvest, soil samples were taken for biochemical and microbiological analyses.

### 4.4. Plants

The choice of spring oilseed rape (Bn) was dictated by the fact that this is a crop grown widely in Europe, especially in Germany, Poland, Lithuania, Latvia, France and Italy [110]. This plant serves to produce edible oils and biofuels. Diesel oil made from oilseed rape played an important role in attaining the goal set by the European Union of reaching 20% of total energy share from renewable energy sources and 10% of RES in transport until the year 2020 [111]. Maize is another leading crop that significantly contributes to the production of bioethanol, with the USA being the main producer thereof. In 2020, 1/3 of farmsteads across the world grew maize and this share is estimated to increase until 2030 by 5%, from 216 to 227 million [112]. Our experiment consisted of two series. In one, 15 seeds of spring oilseed rape (Bn) were sown per pot, whereas in the other 10 maize kernels (Zm) were seeded in each pot. At BBCH 10 stage, the plants were thinned leaving 5 plants of rape and 4 plants of maize in a pot. Both plants were submitted to an assay of the leaf greenness index SPAD (Soil and Plant Analysis Development, SPAD), corresponding with the yielding of plants. The average SPAD index value was determined from eight readings on 5 leaves from each plant before its cutting. Determinations were made with a SPAD 502 Chlorophyll Meter 2900P. Plants were harvested at the full flowering stage (BBCH 59) of oilseed rape and the 6th node stage (BBCH 36) of maize. The aerial parts, same as roots, were dried at 60 °C for 5 days and then weighed. The content of macronutrients was determined in the dry matter of spring oilseed rape and maize aerial parts. N (Kjeldahl’s method; Buchi B-324 distiller, Buchi, Flawil, Switzerland), P (UV–VIS spectrophotometric method; Jenway 6705 UV/VIS spectrophotometer, Jenway LTD, Staffordshire, UK), K (flame photometry method; Jenway PFP 7 flame photometer, Jenway LTD, Staffordshire, UK), Mg and Ca (flame atomic absorption spectrometry (AAS); Mg—atomic absorption spectrophotometer GBC 932AA, GBC Scientific Equipment Australia; Ca—atomic absorption spectrophotometer Agilent 280 FS AA, Agilent Technologies, Mulgrave, Australia) [113]. To this end, the plant material was digested in concentrated H_2_SO_4_ (98%), with 30% solution of H_2_O_2_ acting as an oxidant. Nitrogen (N) was determined by measuring 50 cm^3^ of the mineralizate. The material was titrated against the Tashiro indicator, and the unbound part of sulfuric acid (0.05 mol dm^−3^) was titrated with NaOH solution (0.1 mol dm^−3^). During the determination of phosphorus (P), the extinction of the calibration solutions was measured at a wavelength of 470 nm. The potassium (K) content was determined in 4 cm^3^ of the mineralizate. The determination of the content of magnesium (Mg) and calcium (Ca) by the AAS method was performed at a wavelength of 285.2 nm and 422.6 nm, respectively.

The content of macronutrients was determined in a laboratory accredited by the PCA (Polish Center for Accreditation), at the Regional Chemical and Agricultural Station in Olsztyn (Poland).

### 4.5. Biochemical Analyses

In order to evaluate the condition of soil under the increasing pressure of BPA contamination, all pots had the soil’s biochemical activity determined, including the activity of seven soil enzymes: dehydrogenases (EC 1.1) (μMol triphenyl fomazan kg^−1^ d.m. of soil h^−1^), [104], catalase (EC 1.11.1.6) (Mol O_2_ kg^−1^ d.m. of soil h^−1^), urease (EC 3.5.1.5) (mMol N-NH_4_ kg^−1^ d.m. of soil h^−1^), *β*-glucosidase (EC 3.2.1.21), arylsulfatase (EC 3.1.6.1), acid phosphatase (EC 3.1.3.2) and alkaline phosphatase (EC 3.1.3.1) (mMol p-nitrophenol kg^−1^ d.m. of soil h^−1^) [105], considered to be reliable soil fertility indicators. The following substrates were used in determination of enzyme activity: Deh—2,3,5-triphenyl tetrazolium chloride (TTC), Pal and Pac—disodium4-nitrophenyl phosphate hexahydrate (PNP), Ure—Urea, aqueous solution, Glu—4-nitrophenyl-*β*-D-glucopyranoside (PNG), Aryl—potassium-4-nitrophenylsulfate (PNS), Cat—H_2_O_2_, aqueous solution. The activity of dehydrogenases and five enzymes from the class of hydrolases was determined on a Perkin–Elmer Lambda 25 spectrophotometer (Waltham, MA, USA) with a wavelength range of 190–1100 nm and accuracy of 0.5 nm. The activity of dehydrogenases was determined at a wavelength (λ) of 485 nm; the activity of acid phosphatase, alkaline phosphatase and urease, at 410 nm; the activity of *β*-glucosidase at 400 nm; and the activity of arylsulfatase at 420 nm. The main function of catalase at high concentrations of hydrogen peroxide is to participate in its decomposition to water and oxygen [114]. Values of the activity of catalase was obtained by titration of hydrogen peroxide residues, as described by Borowik et al. [103].

Determinations of the activity of all enzymes were made in three replicates. Based on the results, the biochemical soil fertility index (BA_21_) was calculated.

### 4.6. Microbiological Assays

#### 4.6.1. Determination of Soil Microorganisms

Using the method of serial dilutions, each soil sample underwent the determination of the abundance of five groups of microorganisms: *Arthrobacter* sp. and *Pseudomonas* sp., reflecting the biodegradable potential against organic pollutants [32,115] and counts of organotrophic bacteria (Org), actinomycetes (Act) and fungi (Fun), based on which two indicators, colony development (CD) and ecophysiological diversity (EP), were calculated. The colony forming units of *Arthrobacter* sp. and *Pseudomonas* sp., multiplying on Petri dishes, were counted after 4 days of incubation, whereas colonies of organotrophic bacteria, actinomycetes and fungi were counted for the following 10 days. The composition of microbiological media were as follows: for the *Pseudomonas* sp. (Mulder and Antheunisse medium): peptone 20 g; K_2_HPO_4_ 1.5 g; MgSO_4_·7H_2_O 1.5 g; agar 14.0 g; glycerol 10 cm^3^; H_2_O 1.0 dm^3^; pH 7.2; for *Arthrobacter* sp. (Mulder and Antheunisse): CaH_2_PO_4_ 0.25 g; K_2_HPO_4_ 1.0 g; MgSO_4_·7H_2_O 0.25 g; glycerol 10 cm^3^; agar 14.0 g; H_2_O 1.0 dm^3^; pH 7.0; for organotrophic bacteria (Bunt and Rovira medium): peptone 1.0 g, yeast extract 1.0 g, (NH_4_)_2_SO_4_ 0.5 g, CaCl_2_, K_2_HPO_4_ 0.4 g, MgCl_2_ 0.2 g, MgSO_4_·7H_2_O 0.5 g, Mo salt 0.03 g, FeCl_2_ 0.01 g, agar 20.0 g, soil extract 250 cm^3^, distilled water 750 cm^3^, pH 6.6–7.0; for actinomycetes (Parkinson medium): soluble starch 10.0 g; casein 0.3 g; KNO_3_ 2.0 g; NaCl 2.0 g; K_2_HPO_4_ 2.0 g; MgSO_4_·7H_2_O 0.05 g; CaCO_3_ 0.02 g; FeSO_4_ 0.01 g; agar 20.0 g; H_2_O 1 dm^3^; 50 cm^3^ aqueous solution of nystatin 0.05%; 50 cm^3^ aqueous solution of actidione 0.05%; pH 7.0; and for fungi (Martin medium): peptone 5 g; K_2_HPO_4_ 1.0 g; glucose 10 g; MgSO_4_ 7H_2_O 0.5 g; agar 20.0 g; H_2_O 1 dm^3^; 3.3 cm^3^ aqueous solution of Bengal rose 1%; 25 cm^3^ aqueous solution of aureomycin 0.01%; pH 5.9. All groups of microorganisms were incubated at constant temperature 28 °C. The methodology of all analyses were previously described in Borowik et al. [116]. The number of colony-forming units (cfu) was determined with the help of a colony counter, and all determinations were performed in four replicates.

#### 4.6.2. Isolation of DNA and Bioinformatics Analysis of Bacterial and Fungal Taxa

Extraction and precipitation of genomic DNA from 1 g of soil were achieved with the use of mutanolysin and lysozyme prior to the determination of DNA using a Genomic Mini AX Bacteria+ kit (A&A Biotechnology, Gdańsk, Poland). The subsequent stages consisted of mechanical lysis on a FastPrep-24 device and DNA purification based on the binding of polyphenolic inhibitors of the PCR reaction to appropriate absorption particles obtained on an anti-inhibitor kit. Bacterial DNA was determined with the colorimetric method and verified by Real Time PCR (A&A Biotechnology s. c., Gdańsk, Poland). Amplicon sequencing for all *Bacteria* taxonomic groups was based on the V3–V4 fragment of the 16S rRNA gene. Fungal assemblages were analysed according to the hypervariable region ITS1. All starters used in this part of the experiment as well as PCR settings are presented in Table 4. Illumina MiSeq PE300 (Genomed S.A., Warsaw, Poland) was used for sequencing. Preliminary analysis of the data was performed using a software package MiSeq with MiSeq Reporter (MSR) v2.6 (Illumina, Inc., San Diego, CA, USA). The bioinformatics assay was supported by Qiime software, which relies on the database of reference sequences Greengenes v13_08 (for bacteria) and UNITE v7 (for fungi). The article presents the research results where the OTU richness exceeded 1%. OTU values were employed to determine the diversity of bacteria according to the Shannon–Wiener index (H’) and Simpson index (D) [117]. The following software was used for data visualization: STAMP 2.1.3. [118]; RStudio v1.2.5033 [119] z R project, gplots v3.6.2 [120,121], Venn diagram [122] and Statistica 13.1 [123].

### 4.7. Calculation Methodology and Statistical Data Analysis

Configuration of the research results involved the use of statistical analysis tools from Statistica 13.1. [123]. The following statistical analyses were made: the percentage of the variance of a dependent variable (η^2^) with the variance analysis method ANOVA; multidimensional and exploratory analysis—PCA, displaying the effect of BPA on values of the colony development index (CD) and ecophysiological diversity index (EP), and the content and uptake of macronutrients in aerial plant organs. The Tukey test at P = 0.05 served to identify homogenous variations between enzymes and groups of microorganisms. Differences in the response of enzymes, microorganisms and plants to the applied xenobiotic were evidenced with the tolerance index (TI), which was calculated from the formula:

(1) $${\mathrm{TI}}_{\mathrm{BPA}}=\frac{Y_{\mathrm{BPA}}}{Y_{C}}\;100$$

where: ${\mathrm{TI}}_{\mathrm{BPA}}$—tolerance index of soil enzymes, microorganisms and plants (aerial part and roots) to the increasing levels of BPA soil contamination, (TI < 100—inhibitory effect of BPA; TI > 100—stimulating effect of BPA); Y_BPA_—activity of enzyme, count of microorganisms, and yield of aerial part and roots of a plant in soil submitted to the increasing pressure of BPA contamination; Y_C_—activity of enzyme, count of microorganisms, and yield of aerial part and roots of a plant in control soil, uncontaminated BPA.

The biochemical soil fertility index (BA_21_) was calculated using the formula presented by Wyszkowska et al. [124]:

BA_21_ = Deh + Cat + Pal + Pac + Ure + Glu + Aryl

where: Deh—dehydrogenase, Cat—catalase, Pal—alkaline phosphatase, Pac—acid phosphatase, Ure—urease, Glu—*β*-glucosidase, Aryl—arylsulfatase.

The CD and EP indices were calculated from the following formulas:

$$\mathrm{CD}=\left[\frac{N1}{1}+\frac{N2}{2}+\frac{N3}{3}\ldots \;\frac{N10}{10}\right]\cdot 100$$

where: N_1_, N_2_, N_3_ … N_10—_ratio of grown colonies after 1, 2, 3, …, 10 days.

EP = −Σ(pi × log pi)

where: pi is the number of microbial colonies on a given day divided by the number of all colonies.

In addition, the ratio of the mass of aerial parts to mass of roots of plants (PR) was computed:

$$\mathrm{PR}=\frac{P}{R}$$

where: PR—ratio of the mass of aerial parts to mass of roots of plants; P—dry matter yield of aerial parts; R—dry matter yield of roots.

## 5. Conclusions

Contamination of soil with bisphenol A significantly distorts the microbiological and biochemical soil balance as well as the growth and development of plants. The biotic stress triggered by the presence of BPA in soil caused varied responses among both microorganisms and soil enzymes. Dehydrogenases, urease, acid phosphatase and *β*-glucosidase can be considered reliable indicators of the scale of disturbance of soil homeostasis. BPA significantly inhibited the activity of these enzymes, as opposed to alkaline phosphatase and arylsulfatase. It stimulated the multiplication of *Pseudomonas* sp. and *Arthrobacter* sp. and slowed down the multiplication of fungi. Metagenomic analysis showed that *Proteobacteria* were the dominant phylum of bacteria and *Ascomycota* were the prevalent fungal phylum in soil. The metagenomic analysis also enabled us to distinguish bacteria of the genera *Novosphingobium* and *Sphingomonas*, and fungi of the genus *Penicillium*. As a result, further research directions were set out to define the bioremediation properties of these microorganisms in various types of soils contaminated with phenolic compounds. Bisphenol A disturbs the growth and development of spring rape more strongly than that of maize by interfering with the reduction of the root mass of this plant.

## Figures and Tables

**Table 1:** Tolerance index (TI) of soil enzymes to contamination with BPA.

Dose of BPA mg kg^−1^	Deh	Cat	Pac	Pal	Aryl	Glu	Ure
Soil sown with spring rape (Bn)							
10	282.29 ^a^	106.45 ^b^	113.68 ^a^	114.60 ^b^	107.01 ^b^	105.64 ^a,b^	103.67 ^a^
100	126.31 ^b,c^	94.99 ^b^	105.69 ^a,b^	116.77 ^b^	109.57 ^b^	106.50 ^a,b^	122.43 ^a^
1000	37.88 ^d^	92.09 ^b^	82.00 ^d^	121.48 ^b^	181.09 ^a^	84.77 ^b^	56.56 ^b^
Soil sown with maize (Zm)							
10	146.11 ^b^	94.59 ^b^	103.08 ^b,c^	109.99 ^b^	108.85 ^b^	96.69 ^a,b^	113.20 ^a^
100	135.32 ^b^	95.24 ^b^	93.24 ^c^	113.80 ^b^	109.23 ^b^	110.19 ^a^	100.91 ^a^
1000	88.66 ^c^	132.30 ^a^	75.52 ^d^	161.90 ^a^	168.80 ^a^	86.01 ^b^	31.94 ^c^

Deh—dehydrogenases; Cat—catalase; Ure—urease; Pal—alkaline phosphatase; Pac—acid phosphatase; Glu—*β*-glucosidase; Aryl—arylsulfatase. Homogeneous groups specified in columns denoted with letters (a–d) for each enzyme, depending on the increasing doses of BPA and sowing the soil with plants.

**Table 2:** Tolerance index (TI) of microorganisms in soil contaminated with BPA.

Dose of BPA mg kg^−1^	Ps	Art	Org	Act	Fun
Soil sown with spring rape (Bn)					
10	291.33 ^c^	141.40 ^c^	92.24 ^b,c^	166.32 ^c^	308.55 ^b^
100	919.82 ^a^	611.20 ^b^	141.89 ^b^	269.72 ^a,b^	422.39 ^a^
1000	1008.72 ^a^	1125.87 ^a^	266.36 ^a^	177.48 ^b,c^	68.70 ^c^
Soil sown with maize (Zm)					
10	77.71 ^d^	86.13 ^c^	58.56 ^c^	134.76 ^c^	273.99 ^b^
100	280.03 ^c^	163.15 ^c^	107.83 ^b,c^	150.08 ^c^	311.91 ^b^
1000	718.81 ^b^	185.58 ^c^	132.82 ^b,c^	291.01 ^a^	154.78 ^c^

Org—organotrophic bacteria, Act—actinomycetes, Fun—mold fungi, Ps—*Pseudomonas* sp., Art –*Arthrobacter* sp.; homogeneous groups specified in columns denoted with letters (a–d) were calculated separately for each group of microorganisms, depending on the increasing doses of BPA and sowing the soil with plants (for abbreviations see Figure 1).

**Table 3:** Soil characteristics (biochemical and microbiological properties).

Parameters	Unit	Value	References
Enzyme activity	kg^−1^ d.m. of soil h^−1^		
Deh	μMol TFF	3.996	[104]
Ure	mMol N-NH_4_	0.227	[105]
Cat	Mol O_2_	0.138	
Pal	mMol 4-nitrophenol (PN)	0.675	
Pac		1.989	
Aryl		0.014	
Glu		0.612	
Number of microorganisms	kg^−1^ d.m. of soil		
Org	10*^n^* cfu	42.20	[106]
Act		8.99	[107]
Fun		11.57	[108]
Ps		1.42	[109]
Art		2.63	

Deh—dehydrogenases; Ure—urease, Pal—alkaline phosphatase, Pac—acid phosphatase, Aryl—arylsulfatase, Glu—*β*-glucosidase; Org - organotrophic bacteria, Act—actinomycetes, Fun—mold fungi, Ps—*Pseudomonas* sp., Art—*Arthrobacter* sp., *n*–exponent: 8 for Org, Act, Ps, Art, 6 for Fun.

**Table 4:** PCR conditions for bacterial 16S rRNA genes and fungal ITS1 fragment.

Target Gene	Primers	Reaction Mixture	PCR Conditions
Bacterial 16S rRNA	341F (5′-CCTACGGGNGGCWGCAG-3′) 785R (5′-GACTACHVGGGTATCTAATCC-3′)	10 ng of DNA template, 10 μM primers, Q5 Hot Start High-Fidelity 2× Master Mix	98 °C-30 s//25–35 × (98 °C-5–10 s/50–72 °C-10–30 s/72 °C 20–30 s)//72 °C-2 min
Fungal ITS1	ITS1FI2 (5′-GAACCWGCGGARGGATCA-3′) 5.8S (5′-CGCTGCGTTCTTCATCG-3′)		

## Data Availability

Data are available by contacting the authors.

## Supplementary Materials

The following are available online at https://www.mdpi.com/article/10.3390/ijms222312753/s1.
