# Supplementary material for: Bisphenol A—A Dangerous Pollutant Distorting the Biological Properties of Soil
Source: Int J Mol Sci. 2021 Nov 25;22(23):12753. doi: 10.3390/ijms222312753 (PMC8657726; doi:10.3390/ijms222312753)
Supplement: Supplementary file 1 [file ijms-22-12753-s001.zip › ijms-1451304-supplementary.pdf]

International Journal of *Molecular Sciences*

MDPI

Supplementary

Bisphenol A – A Dangerous Pollutant Distorting the Biological Properties of Soil

Magdalena Zaborowska[1], Jadwiga Wyszkowska[1*], Agata Borowik[1], Jan Kucharski[1]

[1] Department of Soil Science and Microbiology, University of Warmia and Mazury in Olsztyn, Plac Łódzki 3, 10 - 727 Olsztyn, Poland; m.zaborowska@uwm.edu.pl; agata.borowik@uwm.edu.pl, jan.kucharski@uwm.edu.pl
*Correspondence: jadwiga.wyszkowska@uwm.edu.pl; https://orcid.org/0000-0002-2156-3780
Tel.: +48 89 523 49 38 (J.W.)

Table S1. Enzymatic activity in soil contaminated with BPA (kg^{-1} d.m. of soil h^{-1})

Dose of BPA mg kg^{-1} d.m. of soil	Deh (µMol TFF)	Cat (Mol O_2)	Pac	Pal	Aryl	Glu	Ure (mMol N-NH_4)
			(mMol 4-nitrophenol PN)				
			soil sown with spring rape (Bn)				
0	3.772^{d}	0.171^{bcd}	1.925^{d}	0.510^{c}	0.026^{bc}	0.742^{bc}	0.261^{b}
10	10.611^{b}	0.181^{bcd}	2.188^{bc}	0.582^{bc}	0.027^{bc}	0.784^{ab}	0.270^{ab}
100	4.747^{d}	0.161^{de}	2.033^{cd}	0.590^{bc}	0.028^{bc}	0.791^{ab}	0.320^{a}
1000	1.424^{e}	0.156^{e}	1.577^{e}	0.615^{b}	0.046^{a}	0.629^{c}	0.148^{c}
			soil sown with maize (Zm)				
0	9.599^{bc}	0.196^{b}	2.295^{ab}	0.557^{bc}	0.023^{bc}	0.852^{ab}	0.278^{ab}
10	13.995^{a}	0.185^{bc}	2.365^{a}	0.606^{b}	0.024^{bc}	0.823^{ab}	0.315^{ab}
100	12.970^{a}	0.186^{bc}	2.138^{bc}	0.633^{b}	0.016^{c}	0.937^{a}	0.281^{ab}
1000	8.527^{c}	0.259^{a}	1.733^{e}	0.900^{a}	0.038^{ab}	0.732^{bc}	0.089^{d}

Deh – dehydrogenases; Cat – catalase; Ure – urease; Pal – alkaline phosphatase; Pac – acid phosphatase; Glu – *β*-glucosidase; Aryl – arylsulfatase. Homogeneous groups specified in columns denoted with letters (a–e), for each enzyme, depending on the increasing doses of BPA and sowing the soil with plants.

Table S2. The number of microorganisms in soil contaminated with BPA (10^n cfu · kg^{-1} d.m. of soil)

Dose of BPA mg kg^{-1} d.m. of soil	Org	Act	Fun	Ps	Art
			soil sown with spring rape (Bn)		
0	16.250^{de}	6.983^{e}	13.695^{d}	1.413^{c}	1.202^{f}
10	14.429^{e}	11.440^{cd}	42.065^{b}	3.432^{c}	1.606^{ef}
100	22.608^{d}	18.505^{b}	57.798^{a}	10.251^{b}	6.738^{bc}
1000	42.472^{ab}	12.391^{cd}	9.375^{d}	10.724^{b}	12.609^{a}
			soil sown with maize (Zm)		
0	33.722^{c}	9.972^{de}	11.576^{d}	3.643^{c}	5.246^{bcd}
10	19.755^{de}	13.478^{cd}	31.684^{c}	2.828^{c}	4.242^{def}
100	36.358^{bc}	14.918^{bc}	36.087^{bc}	10.245^{b}	7.471^{bc}
1000	44.782^{a}	28.858^{a}	17.907^{d}	25.968^{a}	8.796^{ab}

n–exponent: 8 for Org, Act, Ps, Art; 6 for Fun; Homogeneous groups specified in columns denoted with letters (a–f) were calculated separately for each group of microorganisms, depending on the increasing doses of BPA and sowing the soil with plants.

Table S3. Shannon–Wiener and Simpson indices calculated from the abundance of operational taxonomic unit OTU

Bacterial taxon	Bn	BnBPA	Zm	ZmBPA
	Shannon-Wiener index (H′)			
phylum	1.64	1.51	1.65	1.04
class	2.53	2.42	2.62	1.76
order	2.83	2.89	3.10	2.26
family	3.23	3.03	3.31	2.48
genus	2.94	3.19	3.14	2.69
	Simpson index (D)			
phylum	0.71	0.67	0.70	0.48
class	0.84	0.85	0.86	0.70
order	0.85	0.91	0.92	0.80
family	0.92	0.92	0.93	0.82
genus	0.85	0.93	0.90	0.86

Mold fungus taxon	Bn	BnBPA	Zm	ZmBPA
	Shannon-Wiener index (H′)			
phylum	0.79	1.12	0.68	0.51
class	1.15	1.67	1.18	0.88
order	1.50	2.16	1.80	1.02
family	1.62	2.25	2.06	0.92
genus	1.89	2.32	2.25	0.90
	Simpson index (D)			
phylum	0.41	0.59	0.31	0.21
class	0.50	0.74	0.49	0.35
order	0.62	0.81	0.70	0.36
family	0.62	0.82	0.72	0.31
genus	0.69	0.82	0.71	0.29

Table S4. Average SPAD values on day 59 of spring rape (Bn) and maize (Zm) growth in soil contaminated with BPA

Dose of BPA kg^{-1} d.m. of soil	Spring rape (Bn) BBCH 59	Maize (Zm) BBCH 59
0	36.71[a]	34.79[a]
10	35.23[a]	31.94[a]
100	38.80[a]	33.26[a]
1000	35.27[a]	33.12[a]

Homogeneous groups specified in columns were calculated separately for each plant
